# Measuring thyroid peroxidase antibodies on the day nulliparous women present for management of miscarriage: a descriptive cohort study

**DOI:** 10.1186/1477-7827-11-40

**Published:** 2013-05-14

**Authors:** Mathis Grossmann, Rudolf Hoermann, Claire Francis, Emma J Hamilton, Aye Tint, Tu’uhevaha Kaitu’u-Lino, Kent Kuswanto, Martha Lappas, Ken Sikaris, Jeffery D Zajac, Michael Permezel, Stephen Tong

**Affiliations:** 1 Department of Medicine, Austin Health, University of Melbourne, Studley Road, Heidelberg, Victoria 3084, Australia; 2 Department of Obstetrics and Gynaecology, Mercy Hospital for Women, University of Melbourne, Studley Road, Heidelberg, Victoria 3084, Australia; 3 Melbourne Pathology, 103 Victoria Parade, Collingwood, Victoria 3066, Australia; 4 Translational Obstetrics Group, Department of Obstetrics and Gynaecology, Mercy Hospital for Women, University of Melbourne, Studley Road, Heidelberg, Victoria 3084, Australia

**Keywords:** Thyroid peroxidase antibodies, Miscarriage, Vitamin D, Thyroid autoimmunity

## Abstract

**Background:**

There has been recent evidence suggesting the presence of anti-thyroid peroxidase antibodies (TPOAb) increases the risk of miscarriage, and levothyroxine can rescue miscarriages associated with TPOAb. We propose the most clinically pragmatic cohort to screen for TPOAb are women presenting for management of a missed miscarriage and have never birthed a liveborn. We measured serum TPOAb among nulliparous women presenting for management of miscarriage, and compared levels with women who have had 2 or more livebirths (and never miscarried). Given its potential role in immunomodulation, we also measured Vitamin D levels.

**Methods:**

We performed a prospective descriptive cohort study at a tertiary hospital (Mercy Hospital for Women, Victoria, Australia). We measured TPOAb and Vitamin D levels in serum obtained from 118 nulliparous women presenting for management of miscarriage, and 162 controls with 2 or more livebirths (and no miscarriages). Controls were selected from a serum biobank prospectively collected in the first trimester at the same hospital.

**Results:**

Nulliparous women with 1 or more miscarriages had higher thyroid peroxidase antibody (TPOAb) levels than those with 2 or more livebirths; TPOAb in miscarriage group was 0.3 mIU/L (interquartile range [IR]: 0.2-0.7) vs 0.2 mIU/L among controls (IR 0.0-0.5; p < 0.0001). We confirmed TPOAb levels were not correlated with serum human chorionic gonadotrophin (hCG) concentrations in either the miscarriage or control groups. In contrast, thyroid stimulating hormone, fT3 and fT4 levels (thyroid hormones) either trended towards a correlation, or were significantly correlated with serum hCG levels in the two groups. Of the entire cohort that was predominantly caucasian, only 12% were Vitamin D sufficient. Low Vitamin D levels were not associated with miscarriage.

**Conclusions:**

We have confirmed the association between miscarriage and increased TPOAb levels. Furthermore, it appears TPOAb levels in maternal blood are not influenced by serum hCG levels. Therefore, we propose the day nulliparous women present for management for miscarriage is a clinically relevant, and pragmatic time to screen for TPOAb.

## Background

Miscarriage is the most common complication of pregnancy. Despite this, there are no established treatments that can prevent most cases of sporadic miscarriages. Therefore, any potential therapeutic leads would be of avid interest to clinicians.

An association between circulating thyroid peroxidase antibodies (TPOAb) and miscarriage has been found by a number of observational studies and confirmed by a recent meta-analysis [1] (Thyroid peroxidase is an enzyme that helps produce the thyroid hormones T3 and T4, by liberating iodine so it can be added to tyrosine residues on thyroglobulin). Proposed hypotheses’ to account for this association include direct pathogenicity of thyroid autoantibodies, that the presence of thyroid autoantibodies is a marker for generalized autoimmunity, or thyroid autoantibodies may directly diminish thyroid reserve and increase the risk of miscarriage [2-4].

Of particular clinical relevance, there is evidence treating those with elevated TPOAb levels but otherwise euthyroid (normal thyroid hormone [ie fT3 and fT4] levels) may rescue pregnancies destined for miscarriage. Negro *et al.* reported a randomised trial suggesting administering levothyroxine (a synthetic form of the thyroid hormone T4 that can be taken orally) to women who are TPOAb positive may decrease the risk of miscarriage [5]. The results were quite remarkable, where the risk of miscarriage with TPOab positivity and no treatment was 13.8%, but decreased to only 3.5% with treatment. However, the total cohort in that trial was 110 meaning this finding requires confirmation.

Given the exciting possibility that treating women who are TPOAb positive may prevent cases of sporadic miscarriage (a pregnancy complication that thus far has remained remarkably resistant to any proposed treatments), it may be timely to consider when may be the most clinically pragmatic time to measure TPOAb levels. For example, universal screening of all women of childbearing age, even those who have never been pregnant, may not necessarily be the most appropriate strategy. Universal screening will detect many who are TPOAb positive but were never destined to suffer a pregnancy loss as a result. Thus, it is perhaps not acceptable - or cost effective - to universally screen, where potentially 6% of the entire population [6,7] will be offered treatment.

We propose the appropriate cohort to screen for TPOAb levels may be nulliparous women presenting for management for a miscarriage, for the following reasons: 1) it is clinically convenient as results could be reviewed at the routine 6 week post-procedure check-up (hence management may be streamlined) 2) measuring TPOAb levels among women who have one or more miscarriages but never had a successful liveborn may enrich the number of positive cases, compared to universal screening 3) it may be more clinically acceptable (and more cost effective) to treat women who are TPOAb positive *and* have ever only miscarried, compared to universal screening.

Therefore in this study, we measured TPOAb levels in women nulliparous women presenting for surgical management of their missed miscarriage to: 1) independently verify that TPOAb levels are elevated in association with miscarriage and 2) verify TPOAb levels are not affected by serum hCG and can therefore be validly assessed from a sample obtained during this time.

Serum 25OH-Vitamin D (Vitamin D) deficiency during pregnancy has been linked to a number of adverse maternal and fetal outcomes [8]. Whether vitamin D deficiency increases risk of miscarriage has not been investigated. A link is plausible given that vitamin D deficiency has been linked to increased autoimmunity [9], including thyroid autoimmunity [10,11]. Moreover, the immune system is known to play a critical role in healthy placental implantation [12]. Therefore, we also investigated whether miscarriage is associated with low Vitamin D levels.

## Methods

### Study participants

We conducted a prospective descriptive cohort study at The Mercy Hospital for Women, a tertiary referral centre in Victoria, Australia. Cases were women aged 18 years or older presenting for suction curette as treatment for an incomplete miscarriage diagnosed by ultrasound during the first trimester. Controls were women presenting at their first prenatal visit. We only included controls where the index pregnancy subsequently progressed to a liveborn at term (>37 weeks gestation). Therefore, the control population was defined as women who never miscarried and had at least two successful pregnancies (including the index pregnancy).

The study protocol was approved by the Human Research Ethics Committee at Mercy Hospital for Women and each subject provided written informed consent prior to participation in the study.

### Blood collection and analyses

9 mls of blood was drawn at presentation for curette (cases) and during the first antenatal visit in the first trimester of pregnancy for controls. Thus, cases and controls were matched to the estimated gestation on day of sample collection, where gestation was determined by menstrual dates in miscarriage group and by either menstrual dates and/or ultrasound dating in the control group. Blood was spun at 3000 rpm for 15 minutes at -4°C and the serum was collected and stored at -80°C until assay.

We measured serum levels of the following analytes using a chemiluminescent microparticle immunoassay according to manufacturer’s instructions: TSH, free T4 (fT4), free T3 (fT3) and thyroid peroxidase antibodies (TPOAb) (Architect Anti-TPO assay, Abbot Laboratories, IL, USA). The assay used to quantify thyroid antibody levels has an assay precision of <10% total coefficient of variation. Given different cut-offs (from 5.61 to 12 IU/mL) have been proposed to define to define TPOAb positivity with this particular assay [1,13], we chose *a priori* not to rely on any of these arbitrary cut-offs for our primary analysis but instead, report autoantibody levels as a quantitative parameter.

hCG levels were measured using the Elegance hCG ELISA kit (Bioclone, Australia), with an assay precision of <10% total coefficient of variation (for hCG levels of 4.5 IU/L).

Serum 25OH-Vitamin D (Vitamin D) was measured using the Liaison25OH vitamin D TOTAL (DiaSorin Inc., Stillwater, MN, USA), a direct competitive chemiluminescent immunoassay with an interassay coefficient of variation of 7.0% at 45 nmol/L and 6.3% at 93 nmol/L.

### Data analysis

We used descriptive statistics to report clinical baseline characteristics and analyte values, where quantitative values were expressed as the median and interquartile range (25th - 75th percentiles). Differences between the miscarriage and the control group were analysed by means of nonparametric Wilcoxon rank sum test or chi-square test. Correlations analysis of analytes was performed using spearman’s rank correlation.

## Results

We recruited 118 cases of women presenting for surgical management for a missed miscarriage, diagnosed by ultrasound. From a prospective biobank collection of 1051 samples that were collected at the same hospital contemporaneously, we selected 164 controls, defined as women who never miscarried, had had at least one previous liveborn, and would also go on and successfully deliver a term pregnancy in the index pregnancy (ie at least 2 liveborn and no miscarriage). Serum samples were collected on the day of surgical management for the miscarriage group, and during the mid-first trimester for the control group.

Table 1 shows the baseline characteristics of the 118 cases with miscarriage and the 162 controls with successful term delivery. Of the 118 cases of miscarriage, 69% (n = 81) had had one miscarriage, 22% (n = 26) had two, 6% (n = 7) had three, and 3% (n = 4) had suffered four or more miscarriages. Cases were older (p = 0.028), and there were no differences in ethnicity. The median gestational age at sampling in the missed miscarriage group was 9 weeks’ gestation (ie 9 weeks after their last menstrual period), although fetal demise would have occurred at a variable time prior. However, the data on date of sampling relative to the last menstrual period is relevant as it suggests all those recruited had suffered first trimester miscarriages. Thus, the gestation at sample collection is similar to that for the control group. Those in the control group all delivered at term with normal birthweights (Table 1).

**Table 1:** Baseline characteristics

**Parameter**	**Controls n = 162**	**Cases n = 118**	**P**
Age (years)	31	33	0.028
	(29 – 34)	(28–37)	
Ethnicity			ns
Caucasian	85%	83%	
West South Asian	12%	13%	
North East Asian	1%	2%	
Pacific	1%	2%	
Gravidity	2	2	0.023
	2–2.75	1 - 3	
Parity*	2	0	< 0.0001
	2–2.75	0 - 0	
Gestational age at sampling (weeks)	9	9	ns
	8 - 10	8 - 11	
Gestation Age at delivery (weeks)	39.3	N/A	-
	38.4 - 40.3		
Birthweight (g)	3445	N/A	-
	3120 - 3780		

Values in column 2 and 3 show median and interquartile range (25th to 75th percentile) for each parameter, except ethnicity which is shown in %.

Wilcoxon rank sum test, P > 0.05 is considered non significant (ns).

* Parity includes the index pregnancy.

When examined quantitatively, TPOAb levels were significantly higher in the miscarriage cohort compared to controls (p < 0.0001; Table 2). TPOAb in miscarriage group was 0.3 mIU/L (interquartile range [IR]: 0.2-0.7) vs 0.2 mIU/L among controls (IR 0.0-0.5; P < 0.0001).

**Table 2:** Analyte values

**Parameter**	**Controls n = 162**	**Cases n = 118**	**P**
TPOAb (IU/ml)	0.2 (0–0.5)	0.3 (0.2 - 0.7)	< 0.0001
hCG (IU/L)	107,669 (85,431-139,738)	7,480 (1,627-24,896)	< 0.0001
TSH (mIU/L)	0.66 (0.37 - 1.1)	0.98 (0.73 - 1.44)	< 0.0001
fT4 (pmol/L)	14 (13 – 15)	13.9 (13 – 15)	ns
fT3 (pmol/L)	4.3 (4.0 – 4.7)	3.9 (3.7 - 4.3)	< 0.0001
Vitamin D (nmol/L)	45 (30 – 62)	54 (40 – 65)	0.013

Analyte values are median and interquartile range (25th to 75th percentile).

Wilcoxon rank sum test used for all comparisons.

ns = non significant, where P > 0.05.

When using a cut-off for TPOAb positivity of >5.61 IU/mL (suggested by the manufacturer as this value is >2 standard deviations from the population mean), the incidence of TPOAb positivity in the control population was 14.4% in the miscarriage cohort, compared to 8.8% in the control group, a difference that was not statistically significant (P = 0.18). It is possible this difference may be significant if our study had more numbers given differences in TPOAb titres were highly significant when assessed as a quantitative variable.

At the time of presentation, human chorionic gonadotrophin is likely to be present in the serum, though actively declining. Thus, to validly screen TPOAb titres at the time women present for management of their miscarriage, it would be important to verify levels are not influenced by serum hCG concentrations. Therefore, we performed correlations between TPOAb and hCG in both the miscarriage and control cohorts where the median hCG levels were 7,480 IU/L and 107,669 IU/L respectively. In both statistical comparisons there was no correlation (r values 0.05 cases and -0.01 controls, P ≥ 0.54) between the two analytes suggesting TPOAb are not affected by serum hCG levels (Table 3).

**Table 3:** Analyte correlations with serum hCG

**Parameter**	**Controls**		**Cases**	
	**r =**	**p**	**r =**	**p**
TPOAb (IU/ml)	-0.01	0.87	0.05	0.54
TSH (mIU/L)	-0.25	0.0021	-0.06	0.47
fT4 (pmol/L)	0.14	0.087	0.12	0.21
fT3 (pmol/L)	-0.13	0.12	0.16	0.085

All analytes were correlated with serum hCG using spearman’s rank correlation.

TSH and fT3 levels were significantly higher in the miscarriage cohort compared to controls (Table 2). We also performed correlations between TSH, fT3 and fT4 with serum hCG among cases and controls (Table 3). In contrast to TPOAb, there were some analytes that were either significantly correlated with serum hCG (TSH among the control cohort, r = -0.25, P = 0.0021), or trended towards a correlation that just failed to meet significance (fT3 among the miscarriage cohort, r = 0.16, p = 0.085; or fT4 among controls, r = 0.14, P = 0.087; see Table 3).

Of the entire cohort, only 12% were vitamin D sufficient (defined as vitamin D >75 nmol/L). 37% had vitamin D insufficiency (50–75 nmol/L), 40% mild (25–49 nmol/L) and 11% severe (<25 nmol/L) vitamin D deficiency. Contrary to our hypothesis, absolute vitamin D levels were in fact higher overall in the women who miscarried compared to women with successful term delivery (P = 0.013; Table 2).

## Discussion

While half of all miscarriages are associated with chromosomal abnormalities, the remainder are euploid where miscarriage probably occurs as a result of implantation failure [14]. Thus, a significant proportion of these euploid miscarriages are potentially salvageable [15].

The possibility levothyroxine may salvage some miscarriages caused by TPOAb is a potentially important finding given approximately 6% of women in early pregnancy may be TPOAb positive [6,7]. It is perhaps now timely to consider how such a test could be meaningfully integrated into clinical care. This might inform both future clinical practice and the design of validation trials of levothyroxine treatment for miscarriage in women who are TPOAb positive.

We would propose women who have never previously had a liveborn but have suffered one or more miscarriages may be the appropriate cohort to screen for TPOAb. And we suggest it would be clinically pragmatic to screen women on the day women they present for management of their miscarriage. The TPOAb results can be reviewed at a six-week review where potentially, levothyroxine could be prescribed. The approach is analogous to current clinical management of unexplained stillbirths, where a number of tests are performed during the time women are admitted for induction of labour to empty the uterus.

In this study, we have independently confirmed that higher levels of TPOAb are associated with miscarriage [1-4]. Furthermore, we have confirmed TPOAb levels do not significantly vary with serum hCGs levels suggesting it is may be valid to screen for TPOAb at the time women present for management of their miscarriage. This contrasts with our results for TSH, fT3 and fT4, where we observed correlations with serum hCG levels that were either significant, or just failed to meet significance. This suggests while hCG is able to stimulate the TSH receptor, it does not appear to affect TPOAb levels.

Among the control group, the prevalence of TPOAb was 8.8% (when using the manufacturer’s cut off defined as > 2 standard deviations above the mean). This figure is relatively similar to the prevalence of TPOAb positivity reported in two very large cohort studies. Abbassi-Ghanavati et al. measured TPOAb in serum samples from 17,298 women within the first 20 weeks of gestation and found 6% were TPOAb positive [6]. Similarly, Clearly-Goldman et al. also reported the prevalence of TPOAb positivity among 10,990 samples obtained in the first trimester was 6% [7]. This suggests potentially, our findings may be generalisable to other populations.

Vitamin D deficiency was not associated with an increased risk of miscarriage in our study. In fact, we found vitamin D levels were increased among those who miscarried. This finding was contrary to our hypothesis, which was based on preliminary observations that vitamin D deficiency is associated with thyroid autoimmunity [10,11], as well as other autoimmune diseases in which miscarriage rates may be increased [9]. The biological significance of this finding, if any, is unclear. Given this ran contrary to our hypothesis and the P value just reached significance (P = 0.013), this finding may be a Type I error. Therefore, it firstly requires confirmation in future studies before it merits further consideration.

It is remarkable that even in a region with relatively high levels of sunlight such as southeast Australia, only 12% of pregnant women of predominantly Caucasian descent are vitamin D sufficient. The high prevalence of insufficient vitamin D levels in our cohort is potentially concerning, given that maternal vitamin D deficiency may be associated with a number of adverse pregnancy outcomes [8]. However, the optimal vitamin D level to reduce the risk of pregnancy-associated complications remains unknown.

Our study has some strengths. All samples were collected prospectively and processed in a similar manner at a single centre. The laboratory analyses’ were done in one batch, and by an operator blinded to the sample identities. Finally, we used commercial assays that are used to process samples for clinical care.

## Conclusions

We have shown TPOAb levels are increased in association with miscarriage, compared to women who have had 2 or more livebirths and never miscarried. We also shown TPOAb levels are independent of serum hCG levels and could be screened clinically at the time women present for management of an incomplete miscarriage.

## Abbreviations

fT3: Free triiodothyronine (known as Free T3); fT4: Free thyroxine (known as Free T4); hCG: Human chorionic gonadotrophin; TPOAb: Thyroid peroxidase antibodies; TSH: Thyroid stimulating hormone; Vitamin D: 25OH-Vitamin D.

## Competing interests

The authors declare that they have no competing interests.

## Authors’ contributions

MG and ST conceived and co-ordinated the project, including recruitment, sample analysis, and data interpretation. CF, EH and KK recruited patients. CF, AT and RH prepared the study database and RH performed the statistical analysis. KS, TK and ML performed the assays. ST, MG and RH wrote the first draft of the manuscript. JZ and MP provided further supervision. All authors reviewed the manuscript and either approved it, or provided input.
